# Modification of innate immune responses to *Bordetella pertussis* in babies from pertussis vaccinated pregnancies

**DOI:** 10.1016/j.ebiom.2021.103612

**Published:** 2021-10-11

**Authors:** Thomas F. Rice, Dimitri A. Diavatopoulos, Yanping Guo, Beverly Donaldson, Marielle Bouqueau, Anna Bosanquet, Sara Barnett, Beth Holder, Beate Kampmann

**Affiliations:** a Department of Metabolism, Development and Reproduction (MDR), Lecturer in Maternal and Fetal Health, Imperial College London, Institute of Reproductive and Developmental Biology (IRDB), Hammersmith Campus, London W12 0HS, UK; b Section of Paediatrics, Department of Medicine, Imperial College London, UK; c Section Pediatric Infectious Diseases, Laboratory of Medical Immunology, Radboud Institute for Molecular Life Sciences, Radboud University Medical Center, Nijmegen, the Netherlands; d Radboud Center for Infectious Diseases, Radboud University Medical Center, Nijmegen, the Netherlands; e National Heart and Lung Institute (NHLI), Imperial College London, UK; f The Vaccine Centre, London School of Hygiene and Tropical Medicine, UK; g Vaccines and Immunity Theme, MRC Unit The Gambia at LSHTM, Gambia

**Keywords:** Cytokine, Chemokine, Human, Reproductive immunology, Vaccination

## Abstract

**Background:**

Tetanus, diphtheria, acellular pertussis, inactivated polio (Tdap-IPV) vaccines administered during pregnancy protect young infants from *Bordetella pertussis* (*B. pertussis*) infection. Whilst the impact of maternal Tdap-IPV vaccination on infants’ humoral response to subsequent pertussis immunisation has been investigated, little is known about any impact on innate responses.

**Methods:**

We investigated the immune response to *B. pertussis* in mothers and infants from Tdap-IPV-vaccinated and unvaccinated pregnancies, utilising a whole blood assay and flow cytometric phenotyping of neonatal natural killer (NK) cells, monocytes and dendritic cells. Blood was collected from mother and umbilical cord at birth, and from infants at seven weeks (one week pre-primary pertussis immunisation) and five months of age (one month post-primary pertussis immunisation). 21 mothers and 67 infants were studied.

**Findings:**

Vaccinated women had elevated pro-inflammatory cytokine responses to *B. pertussis*. At birth, babies of vaccinated women had elevated IL-2 and IL-12 responses, elevated classical monocyte proportions, and reduced monocyte and NK cell cytokine responses. The elevated IL-2 response persisted to seven weeks-of-age, when lower IL-10 and IL-13 responses were also seen. One-month post-primary pertussis vaccination, infants from vaccinated pregnancies still had lower IL-10 responses to *B. pertussis*, as well as lower IL-4.

**Interpretation:**

This study suggests that pertussis vaccination during pregnancy impacts infant cellular immune responses, potentially contributing to the modification of antibody responses already reported following primary immunisation against *B. pertussis*.

**Funding:**

National Institute for Health Research Imperial Biomedical Research Centre and IMmunising PRegnant women and INfants neTwork (funded by the GCRF Networks in Vaccines R&D).

**Research in context**

**Evidence before this study**

Animal models of *B. pertussis* infection have demonstrated a key role for the innate immune response in clearance of bacteria and directing an effective adaptive response to infection. There is little evidence regarding innate responses to *B. pertussis* infection following tetanus, diphtheria, acellular pertussis, inactivated polio (Tdap-IPV) vaccination, particularly in the context of vaccination during pregnancy, which has become the foremost tool in preventing pertussis in young infants. Maternal Tdap-IPV vaccination is associated with reduced infant antibody responses to primary pertussis vaccination.

**Added value of this study**

We present evidence that Tdap-IPV vaccination during pregnancy has effects on the maternal immune system beyond boosting maternal antibody levels; vaccinated mothers had increased cytokine responses to *B. pertussis*, compared to unvaccinated mothers. We also show that Tdap-IPV given during pregnancy is associated with modulation of infant immune response to *B. pertussis*. Babies born to Tdap-IPV-vaccinated mothers have an altered distribution of monocytes at birth, and infants from vaccinated pregnancies showed differences in some cytokine responses to *B. pertussis* at birth, seven weeks of age and five months of age.

**Implications of all the available evidence**

Our findings suggest maternal Tdap-IPV may lead to modification of the fetal immune system, in addition to the protection provided by transfer of maternal antibodies. Further studies are required to determine the clinical relevance of these observations, in terms of infant responses to infection or vaccination.

Alt-text: Unlabelled box

## 1 Introduction

*Bordetella pertussis* (*B. pertussis*) is the causative agent of pertussis disease, which has resurged in many countries in recent years, with a disproportionate number of cases and fatalities in infants too young to be vaccinated [1], [2], [3], [4], [5]. Globally, many countries including the United Kingdom, Belgium, the Czech Republic, the United States, Mexico, Argentina, Brazil and Australia have introduced maternal immunisation programmes utilising tetanus, diphtheria, acellular pertussis vaccines with or without inactivated polio (Tdap-IPV and Tdap, respectively) [6], [7], [8], [9]. This vaccine strategy helps protect infants in the first weeks of life through the transfer of antigen-specific maternal antibodies across the placenta during pregnancy. Both maternal Tdap-IPV [8,[10], [11], [12]] and Tdap vaccination [13], [14], [15], [16], have been demonstrated to be both safe and effective in pregnancy. Several studies [17], [18], [19], [20], but not all [21], have demonstrated that maternal Tdap-IPV immunisation during pregnancy blunts the offspring's antibody responses to primary pertussis immunisation. A recent study suggested that maternal Tdap-IPV vaccination has no effect on infant T and B cell responses to *B. pertussis* at birth [22]. The impact of maternal Tdap-IPV on the infant innate immune response remains to be determined.

Innate responses to *B. pertussis* are important in controlling infection in animal models. During infection in mice, natural killer (NK) cells produce interferon (IFN)-γ, which helps restrict *B. pertussis* to the lungs [23] and mice lacking NK cells or the IFN-γ receptor have disseminated, lethal infections [23,24]. Murine dendritic cells (DCs) secrete pro-inflammatory cytokines and chemokines in response *B. pertussis* [25] and in addition, DCs also produce the anti-inflammatory cytokine interleukin (IL)-10 in response to filamentous hemagglutinin (FHA) and lipopolysaccharide (LPS), promoting the induction of regulatory T cells [25,26]. In humans, isolated NK cells produce IFN-γ in response to *B. pertussis* [27] and induction of cytokines that advance T cell responses to infection in monocyte-derived DCs has been shown [28]. These innate responses to *B. pertussis* therefore both contribute to early restriction and innate clearance of *B. pertussis* as well as programming of an effective adaptive response to infection, including the induction of specific T helper cell subsets.

Following maternal inactivated influenza vaccination, influenza-specific effector memory T cells have been observed in cord blood [29] suggesting vaccines given to the mother during pregnancy may prime the fetal immune system *in utero*. In contrast, a study of maternal pertussis vaccination during pregnancy in Brazil found that T cell cytokine responses to *B. pertussis* in cord blood are unaffected by Tdap-IPV vaccination status of the mother [22]. This discrepancy could be due to the distinct immunogenicity induced by the high-dose inactivated flu vaccine versus the Tdap subunit vaccine used in these studies. Indeed, the acellular pertussis components of Tdap-IPV vaccines have been implicated in the modulation of immune responses by dampening early cytokine responses [26,[30], [31], [32]]. Potential priming or dampening effects of Tdap-IPV vaccination during pregnancy requires further investigation.

Pregnancy and early-life are periods of significant immune modulation and vulnerability to infection. It is therefore important to understand if maternal vaccination could impact the newborn's innate immune system. Here, we investigated early cytokine/chemokine responses to the *B. pertussis* bacterium and to Tdap-IPV vaccine antigens in mothers and infants from Tdap-IPV-vaccinated and unvaccinated pregnancies.

## 2 Methods

### 2.1 Vaccines

Pregnant women vaccinated with a tetanus, diphtheria, acellular pertussis, inactivated polio-containing combination vaccine (Tdap-IPV), in line with vaccine policy in the United Kingdom, were recruited to the study between 2014 and 2018 [21]. The vaccine given was initially Repevax® (Sanofi Pasteur, Lyon, France), with a switch to Boostrix-IPV® (GlaxoSmithKline, Wavre, Belgium) after July 2014. Unvaccinated pregnant women were recruited over the same time-period. Infants received three doses of acellular pertussis containing-vaccine (tetanus, diphtheria, acellular pertussis, inactivated polio, Hib) at 8, 12 and 16 weeks; DTaP5-IPV-Hib (Pediacel®; Sanofi Pasteur) or DTaP3-IPV-Hib (Infanrix-IPV-Hib®; GlaxoSmithKline), according to the United Kingdom national immunisation program.^9^ Maternal vaccine contents are as follows: Repevax® contains DTx (2Lf), TTx (5Lf), PTx (2.5µg), FHA (5µg), Prn (3µg), FIM 2 and 3 (5µg) and inactivated poliovirus. Boostrix-IPV® contains DTx (2.5 Lf), TTx (5 Lf), PTx (8µg), FHA (8µg), Prn (2.5µg) and inactivated poliovirus. Infant vaccines contents are as follows: Pediacel® (Sanofi Pasteur, France) contains DTx (25Lf), TTx (10Lf), PTx (20µg), FHA (20µg), Prn (3µg), FIM 2 and 3 (5µg), inactivated poliovirus and Haemophilus influenzae type b polysaccharide (Hib). Infanrix-IPV-Hib® contains DTx (25Lf), TTx (10Lf), PTx (25µg), FHA (25µg), Prn (8µg), inactivated poliovirus and Hib.

### 2.2 Study subjects and sample collection

Women with singleton, uncomplicated term pregnancies booked for maternity care at Imperial College Healthcare NHS Trust were recruited antenatally. Exclusion criteria included multiple pregnancy, chronic diseases including diabetes, maternal autoimmune disease, blood-borne infections (hepatitis B, HIV and syphilis), hypertension, congenital abnormalities, gestational diabetes and pre-eclampsia. Pregnant women that met the inclusion criteria for the study and met none of the exclusion criteria were approached for recruitment. Vaccination status, including the gestation at time of vaccination was recorded at time of recruitment, and again at the time of birth. Pregnant women in our study classed as unvaccinated never received a pertussis vaccine during pregnancy. Blood samples were collected from the mother within 12 h of delivery and from the umbilical cord immediately following birth. Venous blood was collected from infants at seven weeks (one week pre-primary pertussis immunisation) and five months of age (one month post-primary pertussis immunisation).

### 2.3 Ethics

The study was ethically approved by the London-Hampstead Research Ethics Committee (13/LO/1712) and the National Health Service (NHS) Health Research Authority gave approval for the study to take place within Imperial College NHS Trust. Written informed consent was obtained from all women, who also consented for their babies to participate.

### 2.4 Bacteria and antigens

*B. pertussis* (B1917, isolated in 2000) was obtained via the Dutch National Surveillance System from patients with confirmed whooping cough [33]. *B. pertussis* was cultured in Thalen-Ijssel (THIJS) medium and heat-inactivated by incubating for 30 min at 56°C, as previously described [34,35]. Purified *B. pertussis* antigens pertactin (Prn), filamentous hemagglutinin (FHA) and pertussis toxin (Ptx) were a gift from the Centre for Infectious Disease Control, National Institute for Public Health and the Environment (RIVM), the Netherlands [36].

### 2.5 Whole blood bacterial stimulation assay

Whole blood collected into sodium heparin vacutainer® tubes (Becton Dickinson, UK) was diluted 1:10 in RPMI medium supplemented with 10% fetal calf serum (FCS; Thermo Fisher Scientific, UK) and 1% penicillin-streptomycin (Sigma-Aldrich, UK). Blood was stimulated with heat-killed *B. pertussis* in 96 well plates at a final concentration of 5 × 10^5^ colony forming units per 100µl of diluted blood volume, within two hours of collection. Media alone and 1μg/ml LPS (L2137, from *Salmonella Minnesota*, Sigma, UK) served as negative and positive controls, respectively. The concentrations of bacteria and LPS were optimised in control experiments using bloods from non-pregnant volunteers, and then in maternal and cord blood samples to ensure cytokine responses were within the detectable range for each assay. Plates were incubated at 37°C, 5% CO_2_ for 24 h, after which supernatant was removed and stored at -80°C.

### 2.6 Quantification of early cytokines in culture supernatants

Cytokine concentrations in the supernatants from whole blood assays were determined by multiplex assay (Meso Scale Discovery, USA). Briefly, supernatants were diluted 1:10 with assay-specific diluent and 100μl added to plates pre-coated with capture antibodies for ten cytokines/chemokines: IFN-γ, IL-1β, IL-2, IL-4, IL-6, (IL-8), IL-10, IL-12p70, IL-13 and TNF-α. After incubation with shaking for two hours, detection antibodies for each cytokine (except IL-8) were added and further incubated with shaking for two hours. Read buffer was added to each well and plates were read using a MESO QuickPlex SQ 120. Data produced was analysed using Discovery Workbench 4.0. We did not add the detection antibody for IL-8, as this was consistently out of range for detection. Therefore, IL-8 was measured separately by ELISA (IL-8 Human ELISA kit, Invitrogen, UK) following the manufacturer's instructions. Singleplex ELISAs were also used to detect IL-6, IL-1β and IL-10 responses (all Human ELISA kit, Invitrogen, UK) in cord blood in a series of assays where plasma was removed from whole blood prior to stimulation, and replaced with media or pooled cord blood serum. ELISA plates were read at 450nm using a VersaMax Microplate reader (Molecular Devices, UK) and SoftMaxPro 7.

### 2.7 Phenotyping of cord blood innate immune cells

Peripheral blood mononuclear cells (PBMCs) were isolated from fresh whole umbilical cord blood using Histopaque (Sigma-Aldrich, UK) and cryopreserved in FCS supplemented with 10% dimethyl sulfoxide (Sigma-Aldrich, UK). Cryopreserved PBMCs were rapidly thawed and live cells counted by trypan blue exclusion. Aliquots with less than 80% viability were discarded. PBMCs were added to 96-well round-bottom culture plates at 4 × 10^5^ cells/well in 200µl RPMI (with 10% FCS, 1% penicillin-streptomycin). Cells were rested overnight at 37°C, 5% CO_2_, and then stimulated with heat-killed *B. pertussis* and a pertussis antigen cocktail consisting of pertactin (2 μg/ml), FHA (1 μg/ml) and pertussis toxin (1 μg/ml) for four hours [37]. Optimisation experiments had shown that stimulation of PBMC for a longer period of 18 h resulted in limited ability to detect intracellular cytokine responses. Cultured cells were incubated with 20mM Ethylenediaminetetraacetic acid (EDTA) on ice for 20 min to remove any cells that became adhered to the tissue culture plate surface during stimulation. Cells were washed with PBS and stained with the Zombie Aqua™ Fixable Viability Kit for 15 min following the manufacturer's instructions (BioLegend, UK). Commercially validated antibodies for cell surface and intracellular staining were purchased from BioLegend (UK) unless stated otherwise and were titrated prior to use (**Supplementary Table 1**). No additional validation was performed in-house. Cultured cells were washed and stained for 20 min to identify T cells (CD3Alexa Fluor® 700, SK7, #344822), B cells (CD19 PerCP/Cy5.5, HIB19, #302230), NK cells (CD16 PE/Cy7, B73.1, #360708; CD56 Brilliant Violet 711™, HCD56, #318336; CD107a Brilliant Violet 421™, H4A3, #328626), monocytes (HLA-DR APC/Fire™ 750, L243, #307658; CD16 PE/Cy7, B73.1, #360708; CD14 Brilliant Violet 605™, M5E2, #301834) and DCs (HLA-DR APC/Fire™ 750, L243, #307658; CD123 Brilliant Violet 785™, 6H6, #306032; CD11c PE/Dazzle™ 594, 3.9, #301642).

PBMCs were fixed (4% paraformaldehyde, BioLegend, UK) for 20 min at room temperature, and permeabilized (permeabilization wash buffer, BioLegend, UK). Intracellular cytokine staining utilising antibodies conjugated to PE for IFN-γ (B27, #506506), IL-6 (MQ2-13A5, #501107) and TNFα (MAb11, #502908), and conjugated to APC for IL-10 (JES3-9D7, #501409), IL-1β (8516, Invitrogen, #MA5-23597), and IL-12 (C11.5, #501809), was then performed. Fluorescence minus one (FMO) controls were included for all cytokines to ensure accurate gating of positive populations. Samples were acquired on a BD LSRFortessa Flow Cytometer and data analysed with FlowJo, Version 10 (Tree Star, USA) and Cytobank (Cytobank Inc, USA). A hierarchical gating strategy was used to identify innate cell populations and cytokine producing cells (**Supplementary Fig. 1**), excluding doublets and dead cells [38]. T cells (CD3^+^) and B cells (CD19^+^) were gated out prior to innate immune cell subsetting. NK cell subsetting identified cytokine producing (CD56^bright^CD16^−^) and cytotoxic (CD56^dim^CD16^+^) NK cells. Monocytes were identified by expression of HLA-DR and subsetted as classical (CD14^++^CD16^−^), intermediate (CD14^++^CD16^+^) and non-classical (CD14^+^CD16^++^). HLA-DR-expressing plasmacytoid and myeloid dendritic cells were identified by expression of CD123 and CD11c, respectively.

### 2.8 Statistical analysis

The study was a prospective, observational cohort study that was not randomised nor blinded. A sample size calculation at each time point was not performed. Maternal cytokine concentrations produced in response to *B. pertussis* in Tdap-IPV-vaccinated and unvaccinated groups were compared by Mann-Whitney U test or Student's t test, depending on the distribution of the data. For analysis of infant responses, cytokine concentrations were log transformed (natural log; *Y*=Ln(Y)) to achieve normality, and a random intercepts linear regression model used to analyse the impact of maternal Tdap-IPV on infant cytokine concentrations measured by ELISA/multiplex immunoassay at birth, seven weeks and five months of age, assuming that the data are missing completely at random. The model included the main effects of time and vaccination status, and a time by vaccination interaction. The model accounted for variation in the random intercepts of the individuals. The frequency of each monocyte subset (classical, intermediate, non-classical) within the monocyte population was analysed by 2-way ANOVA (variables: vaccination status and *in vitro* treatment). Cytokine levels measured by flow cytometry in cord PBMCs were analysed by Student's t-test or Mann-Whitney *U* test, depending on the distribution of data. The Friedman test was used to analyse cytokine responses to *B. pertussis* in the absence of blood plasma compared to responses in the presence of plasma. A *P* value <0.05 was considered statistically significant. The random intercepts model was performed using Stata version 16, all other analyses were conducted using GraphPad Prism v8.

### 2.9 Role of the funding source

This article is independent research funded by the National Institute for Health Research (NIHR) Imperial Biomedical Research Centre (BRC) and the IMmunising PRegnant women and INfants neTwork (IMPRINT) funded by the GCRF Networks in Vaccines Research and Development, which was co-funded by the MRC and BBSRC. Neither of these funders had a role in study design; in the collection, analysis, and interpretation of data; in the writing of the report; nor in the decision to submit the paper for publication.

## 3 Results

### 3.1 Study subjects

In total, samples from 21 mothers and 67 infants were included in the study. Samples from vaccinated and unvaccinated pregnancies were collected over similar time periods (**Supplementary Tables 2–6**). Due to the unpredictable timing of sample collection and the need for the whole blood assay to be run on fresh blood, the majority of infants were not included at every time point. Thus, missingness was not related to the outcome measures. Overall, the whole blood assay dataset consisted of 48 infants (40 at one timepoint, six infants at two timepoints, and two infants at all three time points; **Supplementary Fig. 2**). Clinical data for maternal and infant samples used in the whole blood assay are shown in **Supplementary Table 2–5**. Other than Tdap-IPV vaccination status, the two groups were matched for all other variables, including age, BMI, ethnicity, parity, gravidity, except for a higher rate of maternal influenza vaccination for the samples run on seven-week-old infants born to Tdap-IPV-vaccinated mothers (*p*=0.024, Fisher's exact test). For the flow cytometry assay, cord blood samples from 20 individuals were included (one of which was also used in the whole blood assay). Clinical data for the pregnancies from which cord samples were used in the flow cytometry assay are described in **Supplementary Table 6**. There were no significant differences between the groups for any variables, except for Tdap-IPV vaccination status.

Following the switch from Repevax to Boostrix-IPV, we recorded the vaccine brand administered to our recruits based on patient recall. The majority (48/52) women had received Boostrix-IPV, with three women for whom the vaccine brand was unknown. Three of these pregnancies were in the group from which we obtained 5-month-old infant vaccine samples, and one in the group from which we performed flow analyses on cord PBMC. For these four patients, vaccination was delivered at three, four, six and seven months after the advised switch to Boostrix-IPV.

### 3.2 Tdap-IPV vaccination during pregnancy is associated with elevated maternal cytokine responses to *B. pertussis*

**Fig. 1:**
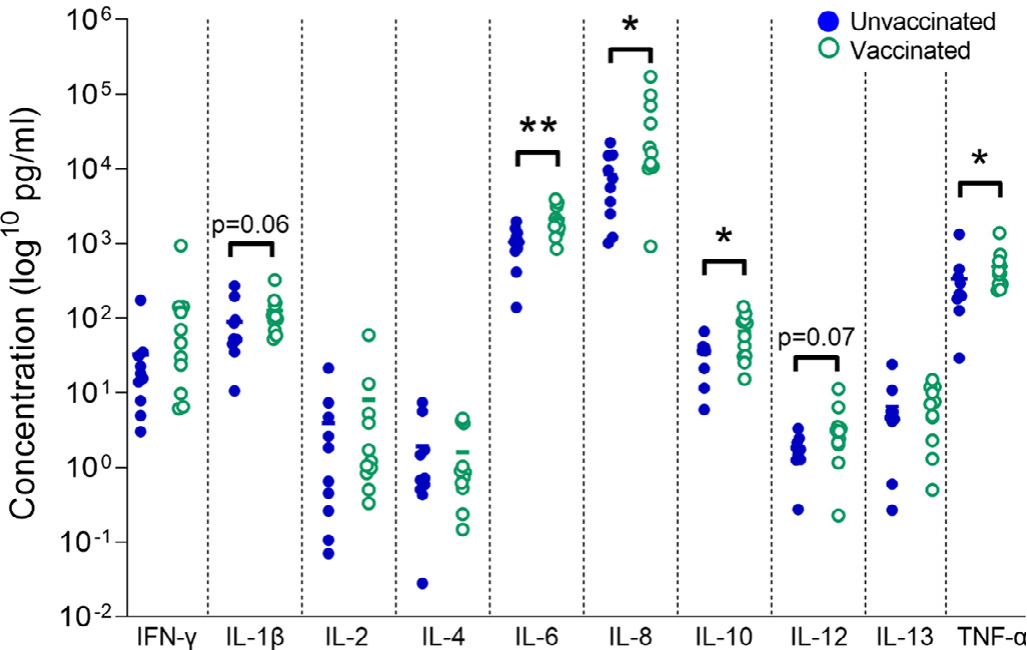
*Impact of maternal Tdap-IPV vaccination on maternal innate cytokine responses to B. pertussis.* Maternal blood was treated for 24 h with heat-killed *B. pertussis* in duplicate and cytokines were quantified in supernatants in singlicate. Data points represent cytokine concentrations plotted on a log10 scale. Unvaccinated *n*=10 (filled blue circles); vaccinated *n*=11 (open green circles). Vaccinated and unvaccinated groups were compared by Student's t test (normal distribution; IL-6 and IL-10) or Mann-Whitney U test (non-normal distribution; all others); * *p*<0.05, ** *p*<0.01. (For interpretation of the references to color in this figure legend, the reader is referred to the web version of this article.)

To investigate whether Tdap vaccination modulates the innate immune response to the causative bacterium of pertussis disease, maternal blood from vaccinated and unvaccinated pregnancies was incubated for 24 h with heat-killed *B. pertussis* bacteria and cytokine release measured by multiplex immunoassay or ELISA. LPS served as a positive control for the experiment, being a potent activator of innate immune responses, primarily through toll-like receptor 4 (TLR-4). IL-6, IL-8, IL-10 and TNF-α responses were all significantly higher in Tdap-IPV-vaccinated women, compared to unvaccinated women (IL-6 866, 95% CI [318, 1930], *p*<0.01, Student's t test; IL-8 9980, 95% CI [1111, 60922], *p*=0.03, Mann-Whitney U test; IL-10 21, 95% CI [-5.7, 68], *p*=0.03, Student's t test; TNF-α 191, 95% CI [31, 358], *p*=0.04, Mann-Whitney U test; Fig. 1). There was a non-significant increase in the IL-1β and IL-12 response in Tdap-IPV-vaccinated women (IL-1β 53, 95% CI [-0.8, 97], *p*=0.06, Mann-Whitney U test; IL-12 1.4, 95% CI [-0.04, 2.1], *p*=0.07, Mann-Whitney U test; Fig. 1). IFN-γ, IL-2, IL-4 and IL-13 responses were unaffected by maternal Tdap vaccination.

### 3.3 Maternal Tdap-IPV vaccination modifies infant cytokine responses to *B. pertussis*

**Fig. 2:**
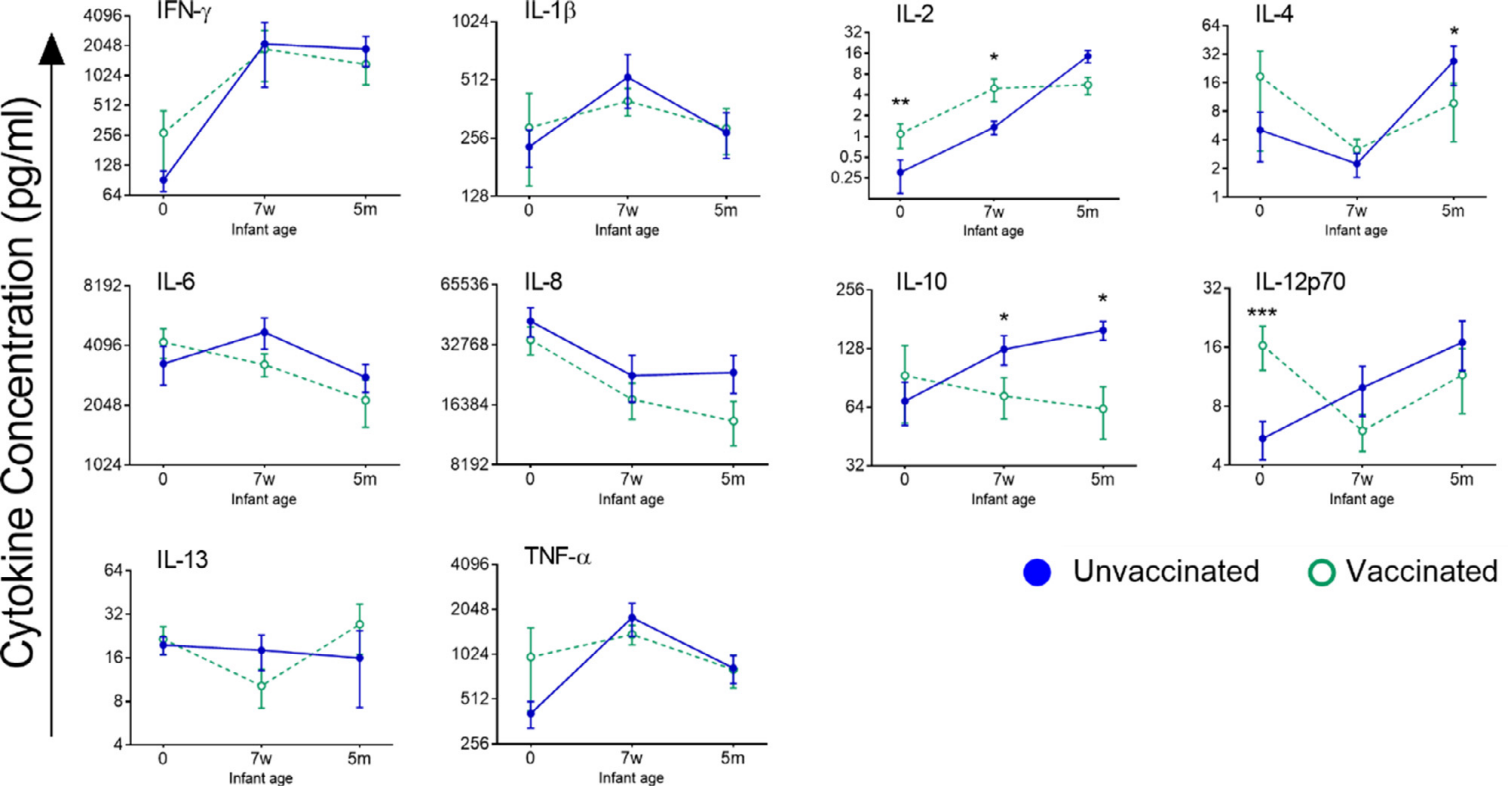
*Impact of maternal Tdap-IPV vaccination on infant innate cytokine responses to B. pertussis.* Blood from the umbilical cord (0) and from infants at seven weeks (7w; pre-primary pertussis immunisation) and five months of age (5m; post-primary pertussis immunisation) was treated with *B. pertussis* for 24 h in duplicate and cytokine concentrations were quantified in supernatants in singlicate. Data points represent cytokine concentrations plotted on a log2 scale and error bars represent the standard error mean. Unvaccinated: cord *n*=10, 7w *n*=10, 5m *n*=7 (filled blue circles and solid line). Vaccinated: cord *n*=11, 7w *n*=10, 5m *n*=10 (open green circles and dashed line). Random intercepts model; * *p*<0.05, ** *p*<0.01, *** *p*<0.01. (For interpretation of the references to color in this figure legend, the reader is referred to the web version of this article.)

To determine the impact of maternal Tdap-IPV on infant cellular responses to *B. pertussis,* cord blood, and blood from 7-week-old and 5-month-old infants was incubated with *B. pertussis* as for maternal blood (Fig. 2**;** Table 1). LPS served as a positive control, as before. At birth, IL-2 and IL-12 responses were significantly higher in infants from Tdap-IPV-vaccinated pregnancies, compared to the unvaccinated group (IL-2 1.5, 95% CI [0.55, 2.47], *p*<0.01; IL-12p70 1.29, 95% CI [0.56, 2.02], *p*<0.001, random intercepts model). At seven weeks of age (prior to pediatric pertussis vaccination), infants from Tdap-IPV-vaccinated pregnancies still had significantly elevated IL-2 responses to *B. pertussis*, compared to infants from unvaccinated pregnancies (0.99, 95% CI [0.02, 2.00], *p*=0.045, random intercepts model), but IL-12 was no longer significantly different (*p*=0.088). In addition, IL-10 and IL-13 responses were decreased in the infants from Tdap-IPV-vaccinated pregnancies, compared to those from unvaccinated pregnancies (IL-10 -0.57, 95% CI [-1.11, 0.03], *p*=0.04; IL-13 -1.07, 95% CI [-2.00, -0.15], *p*=0.02; random intercepts model; Fig. 2**;** Table 1). At five months of age (one month after completion of pediatric pertussis vaccination), the significantly reduced IL-10 response to *B. pertussis* was still seen in infants from Tdap-IPV-vaccinated pregnancies, as well as lower IL-4 (IL-4 -1.37, 95% CI [-2.53, -0.22], *p*=0.02; IL-10 -0.60, 95% CI [-1.22, -0.05], *p*=0.03; random intercepts model; Fig. 2**;** Table 1). All other cytokines (IFN-γ, IL-1β, IL-6, IL-8, IL-13 and TNF-α) were unaffected by maternal Tdap vaccination.

**Table 1:** *Impact of maternal Tdap-IPV on infant cytokine responses to B. pertussis.* Whole blood cytokine responses to *B. pertussis* in infants from Tdap-IPV-vaccinated and unvaccinated pregnancies at birth, seven weeks and five months of age were measured by multiplex cytokine assay/ELISA. Table shows the predicted means of log-transformed outcomes, and the results of these when used in a random intercepts linear regression model to compare cytokine responses in infants from Tdap-IPV-vaccinated and unvaccinated pregnancies at each time point. Positive contrasts denote higher responses in the vaccinated group compared to the unvaccinated group, and negative coefficients denote lower responses. SE; standard error, 95%CI; 95% confidence intervals, *p* values <0.05 indicated in bold.

Age	Cytokine	Unvaccinated	Vaccinated	Contrast (SE)	95%CI	*P* value
Birth	IFN-y	4.66	4.38	-0.27 (0.54)	-1.33, 0.78	0.6103
	IL-1β	5.47	5.08	-0.39 (0.31)	-1.00, 0.22	0.2129
	IL-2	-2.13	-0.62	1.51 (0.49)	0.55, 2.47	**0.0021**
	IL-4	0.83	1.10	0.27 (0 .53)	-0.76, 1.30	0.6048
	IL-6	7.91	8.23	0.31 (0.24)	-0.15, 0.78	0.1891
	IL-8	199.38	179.57	-19.81 (23.15)	-65.18, 25.56	0.3922
	IL-10	4.03	4.07	0.05 (0.29)	-0.51, 0.61	0.8646
	IL-12	1.27	2.56	1.29 (0.37)	0.56, 2.02	**0.0005**
	IL-13	2.60	2.88	0.28 (0.48)	-0.66, 1.22	0.5627
	TNF-a	5.98	5.98	0.001 (0.31)	-0.61, 0.61	0.9969
7 weeks	IFN-y	6.24	6.45	0.21 (0.52)	-0.81, 1.23	0.6853
	IL-1β	5.84	5.90	0.06 (0.31)	-0.55, 0.66	0.8541
	IL-2	0.00	1.00	0.99 (0.50)	0.02, 2.00	**0.0451**
	IL-4	0.41	0.66	0.25 (0.54)	-0.80, 1.30	0.6380
	IL-6	8.30	7.99	-0.32 (0.24)	-0.78, 0.15	0.1877
	IL-8	133.92	119.98	-13.94 (23.34)	-59.68, 31.80	0.5503
	IL-10	4.56	3.98	-0.57 (0.28)	-1.11, 0.03	**0.0384**
	IL-12	1.68	1.08	-0.61 (0.37)	-1.31, 0.09	0.0880
	IL-13	2.46	1.39	-1.07 (0.47)	-2.00, -0.15	**0.0226**
	TNF-a	7.27	7.11	-0.16 (0.32)	-0.78, 0.46	0.6091
5 months	IFN-y	7.10	6.77	-0.32 (0.56)	-1.42, 0.78	0.5658
	IL-1β	5.21	5.35	0.14 (0.33)	-0.52, 0.79	0.6827
	IL-2	2.47	1.43	-1.05 (0.55)	-2.12, 0.027	0.0560
	IL-4	2.62	1.24	-1.37 (0.59)	-2.53, -0.22	**0.0199**
	IL-6	7.79	7.42	-0.37 (0.26)	-0.88, 0.14	0.1553
	IL-8	138.45	106.91	-31.54 (25.57)	-81.65, 18.56	0.2173
	IL-10	4.50	3.86	-0.64 (0.30)	-1.22 - -0.05	**0.0321**
	IL-12	2.31	1.96	-0.36 (0.38)	-1.10 - 0.39	0.3512
	IL-13	2.13	2.32	0.19 (0.51)	-0.81 - 1.19	0.7110
	TNF-a	6.58	6.48	-0.10 (0.35)	-0.78 - 0.58	0.7726

To determine the contribution of antigen-specific maternal IgG present on cytokine responses in infants, cord blood responses were investigated in the absence of plasma. The removal of plasma from cord blood resulted in significant reductions in the IL-1β (88%, *p*<0.0001), IL-6 (75%, *p*<0.001) and IL-10 (76%, *p*<0.01) responses to *B. pertussis* (Friedman test; **Supplementary Fig. 3**). However, these responses were restored by replacement with cord blood serum from either unvaccinated or Tdap-IPV-vaccinated pregnancies (*p*>0.05), suggesting that the observed differences in cytokine responses between the treatment groups cannot be attributed to the presence of vaccination-induced maternally-derived pertussis-specific antibodies.

### 3.4 Tdap-IPV vaccination during pregnancy is associated with altered distributions of monocyte populations in infants at birth

**Fig. 3:**
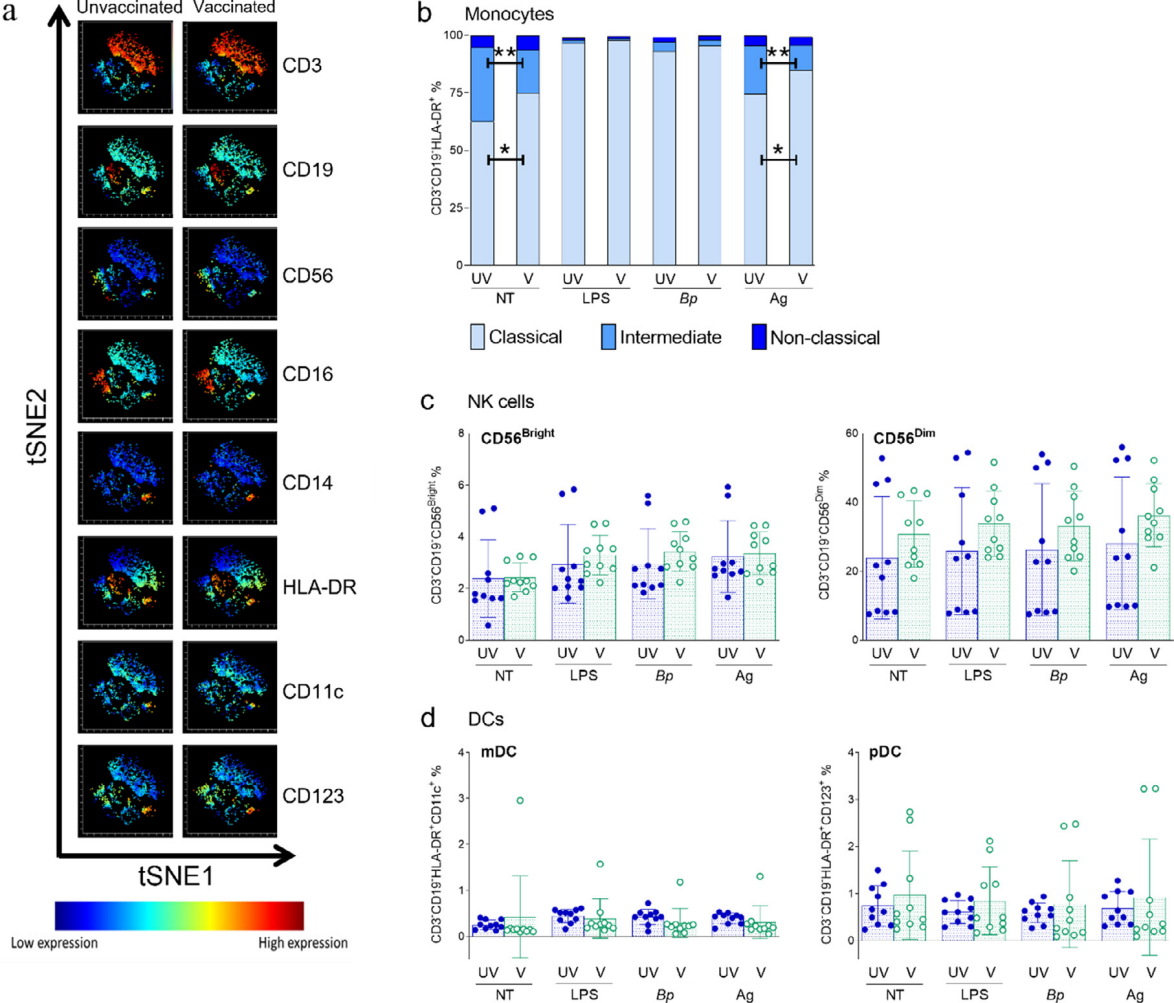
*Modulation of cord blood innate cell populations by maternal Tdap.* Cord peripheral mononuclear cells were stimulated for four hours with *B. pertussis* (Bp) or acellular pertussis vaccine antigens (Ag) and innate cell populations identified by flow cytometry. (a) tSNE analysis of the expression of surface markers on cord peripheral mononuclear cells from unvaccinated and Tdap-IPV-vaccinated pregnancies. (b) Monocyte subsets were identified by the expression of CD14 and CD16 and compared between groups and treatment by 2-way ANOVA. (c) NK cell subsets were identified by the expression of CD56 and CD16. In CD56^Bright^ NK cells, vaccinated and unvaccinated groups were compared by Student's t test (normal distribution; NT) or Mann-Whitney U test (non-normal distribution; all others) and no significant differences were observed between groups. In CD56^Dim^ NK cells, vaccinated and unvaccinated groups were compared by Mann-Whitney U test and no significant differences were observed groups. (d) DC subsets were identified by the expression of CD11c and CD123. In mDCs, vaccinated and unvaccinated groups were compared by Mann-Whitney U test and no significant differences were observed groups. In pDCs, vaccinated and unvaccinated groups were compared by Student's t test (normal distribution; NT, LPS and Bp) or Mann-Whitney U test (non-normal distribution; Ag) and no significant differences were observed between groups. Error bars represent the standard deviation from the mean. For all analyses unvaccinated and Tdap-IPV groups *n*=10. UV: unvaccinated; V: vaccinated; NT: no stimulation; Bp: *B. pertussis*; LPS: lipopolysaccharide; Ag: antigen; * *p*<0.05.

In order to compare innate cell populations in infants born to unvaccinated and Tdap-IPV-vaccinated women, PBMCs from cord blood were stimulated with heat-killed *B. pertussis* as before, as well as a mixture of pertussis antigens contained in the Tdap-IPV vaccine administered to mothers (pertussis toxin, filamentous haemagglutinin and pertactin) and then analysed by flow cytometry. LPS served as a positive control. CD3^+^ T cells and CD19^+^ B cells were gated out and three populations of monocytes were identified as classical (CD14^++^CD16^−^), intermediate (CD14^++^CD16^+^) and non-classical (CD14^+^CD16^++^; Fig. 3**a, Supplementary Fig. 1**). PBMCs from babies born to Tdap-IPV-vaccinated women contained significantly elevated proportions of classical monocytes (75% of total monocyte population) compared to those born from unvaccinated pregnancies (62%, *p*=0.01, 2-way ANOVA; Fig. 3**b**). Similar differences were also observed following vaccine-antigen stimulation (85% versus 74%, *p*=0.01, 2-way ANOVA; Fig. 3**b**), but not following *B. pertussis* nor LPS stimulation. In parallel, there was a reduction in the proportion of intermediate monocytes in PBMCs from infants born to Tdap-IPV-vaccinated women, compared to unvaccinated (19% versus 32%, *p*<0.01, 2-way ANOVA). This difference was again seen following antigen stimulation (11% versus 21%, *p*<0.01, 2-way ANOVA), but not with bacteria or LPS stimulation.

At birth, no significant differences were observed between the treatment groups for neonatal CD56^Bright^ or CD56^Dim^ NK cells under any of the stimulation conditions (*p*>0.05; Fig. 3**a,c**). Tdap-IPV vaccination also did not significantly alter the proportions of myeloid (CD11c^+^) nor plasmacytoid (CD123^+^) dendritic cells (*p*>0.05; Fig. 3**a,d**).

### 3.5 Tdap-IPV vaccination during pregnancy alters cytokine production from monocytes and NK cells in infants at birth

**Fig. 4:**
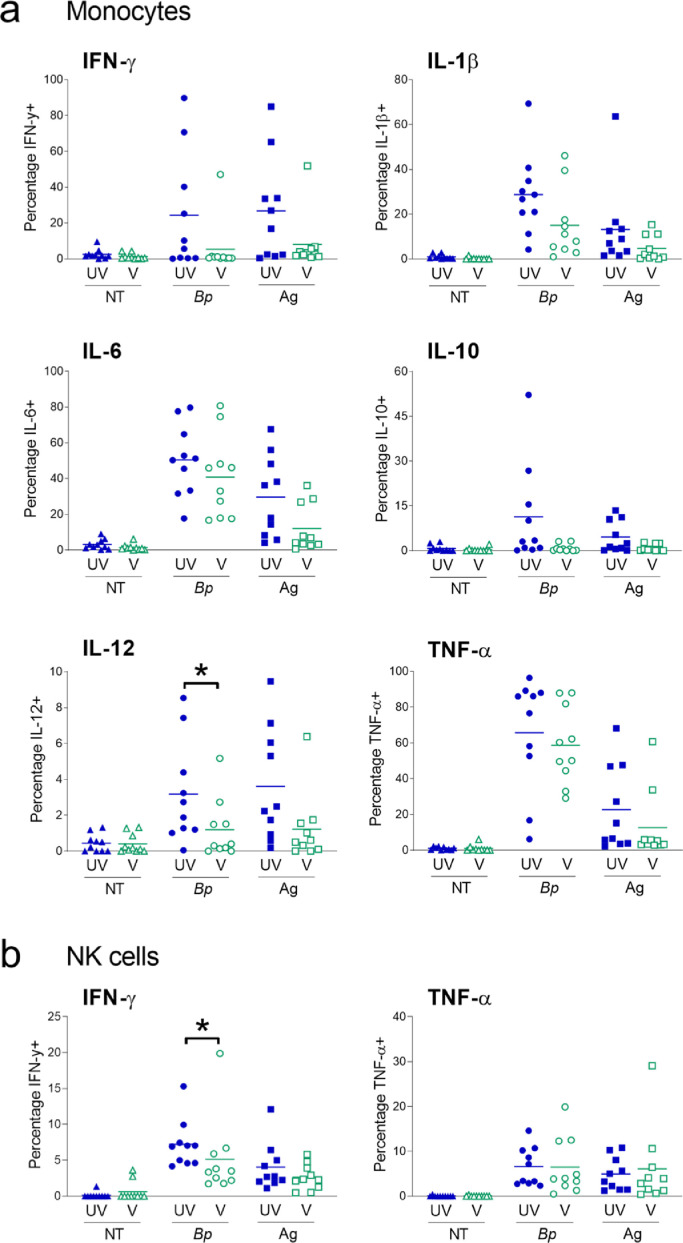
*Modulation of cord blood monocyte and NK cell cytokine levels by maternal Tdap-IPV.* Cord peripheral mononuclear cells were stimulated for four hours with *B. pertussis* (Bp) or acellular pertussis vaccine antigens (Ag) and cytokine levels determined by flow cytometry. (a) In classical monocytes vaccinated and unvaccinated groups were compared by Student's t test (normal distribution; IL-6 and IL-1β) or Mann-Whitney U test (non-normal distribution; all others). (b) In CD56^Bright^ NK cells vaccinated and unvaccinated groups were compared by Mann-Whitney U test. For all analyses unvaccinated and Tdap-IPV groups *n*=10; UV: unvaccinated; V: vaccinated; NT: no stimulation; *Bp*: B*. pertussis*; Ag: antigen; * *p*<0.05.

In order to evaluate the impact of maternal Tdap-IPV on infant innate immune cell function, IFN-γ, IL-1β, IL-6, IL-10, IL-12 and TNF-α production per cell subset were investigated by intracellular cytokine staining following *in vitro* stimulation with *B. pertussis* and Tdap-IPV vaccine antigens in cord blood PBMC (Fig. 4). Maternal Tdap-IPV vaccination did not significantly impact the levels of cytokines in cord blood intermediate and non-classical monocytes (**Supplementary Fig. 4**). This was also the case for classical monocytes, with the exception of IL-12 which was reduced in the Tdap-IPV-vaccinated group, compared to the unvaccinated group (-2.0, 95% CI [-4.0, 0.1], *p*=0.03, Mann-Whitney U test; Fig. 4**a**). In CD56^Bright^ NK cells, a 1.4-fold lower IFN-γ response to *B. pertussis* was observed in infants born to Tdap-IPV-vaccinated women, compared to unvaccinated (-3.5, 95% CI [-5.1, -0.7], *p*=0.02, Mann-Whitney U test; Fig. 4**b**). There were no significant differences in proportion of NK cells positive for TNF-a. Cytokine levels in myeloid and plasmacytoid DCs were generally low in response to all treatments, regardless of vaccination status. There was an increase in TNF-α in the unvaccinated group in response to Tdap-IPV vaccine antigens in myeloid DCs (-6.6, 95% CI [-12.2, 0.1], *p*=0.03, Mann-Whitney U test; **Supplementary Fig. 5**). Tdap-IPV vaccination did not impact cytokine levels in myeloid nor plasmacytoid DCs, whether cells were left untreated or were exposed to *B. pertussis* or Tdap-IPV vaccine antigens, for all other cytokines (**Supplementary Fig. 5**). Whilst some cytokines were also found in T cells and B cells, no significant differences were observed between unvaccinated and Tdap-IPV-vaccinated groups in response to *B. pertussis* nor the Tdap-IPV vaccine antigens (all *p*>0.05; **Supplementary Fig. 6**).

## 4 Discussion

Pertussis vaccination during pregnancy boosts anti-pertussis antibody levels in both mothers and their offspring, and prevents pertussis disease in infants [17], [18], [19], [20], [21]. Several studies have demonstrated that Tdap/Tdap-IPV vaccination in pregnancy is associated with reduced humoral responses to pediatric pertussis vaccination in the offspring [17], [18], [19], [20]. Whether there is also any impact of maternal pertussis vaccination on infant cell-mediated immunity is largely unknown. The only study published to date looked at cord blood B cell, T cell and whole blood cytokine responses to *B. pertussis* and found no effect of maternal Tdap on any of these parameters [22]. Here, we have assessed the impact of maternal Tdap-IPV on cellular responses to *B. pertussis* at birth and infancy; both before and after receipt of paediatric pertussis vaccination. We found that Tdap-IPV vaccination in pregnancy is associated with modulation in cytokine responsiveness in both women and their babies, and altered phenotype and function of infant innate immune cell responses to *B. pertussis.* We propose that these changes to cell-mediated immunity may contribute to the altered humoral response to pertussis vaccines seen in babies from Tdap-IPV-vaccinated pregnancies.

Our findings suggest that recently Tdap-IPV-vaccinated pregnant women may be primed to mount a greater innate cytokine response to *B. pertussis*. Tdap-IPV vaccination was associated with elevated *in vitro* whole blood cytokine responses to *B. pertussis* in maternal blood at birth, with higher production of IL-6, IL-8, IL-10 and TNF-α; cytokines associated with both pro- and anti-inflammatory responses. Pertussis antigens contained in the Tdap-IPV vaccine have been previously shown to induce cytokine production *in vitro*; FHA induces TNF-α production in human monocyte-derived macrophages [39] and stimulates the production of IL-10 and IL-6 in mouse macrophages and DCs [26,30]. In addition, this effect could be due to the presence of pertussis-specific T cells in Tdap-IPV-vaccinees, given the role for T cells in regulating early innate inflammatory responses [40], [41], [42], [43].

The offspring of Tdap-IPV-vaccinated women also exhibited altered cell-mediated immune responses to *B. pertussis.* Babies from Tdap-IPV pregnancies had elevated IL-2 and IL-12p70 whole blood responses to *B. pertussis* at birth. The production of IL-12 by innate cells during *B. pertussis* infection enables the polarisation of the T cell response, to the T helper type 1 (Th1) subtype [44]. IL-2 acts primarily as a T cell growth factor, essential for the proliferation and survival of T cells, and is mainly produced by activated CD4^+^ T cells, also in response to *B. pertussis* [45,46]. At birth, all other whole blood cytokine responses were unchanged, suggesting a circumscribed modulation of the neonatal immune system by maternal Tdap-IPV.

In infants at birth, we additionally characterised innate immune cell subsets and their cytokine response by flow cytometry. Monocytes transition from classical, to intermediate, to non-classical, with the non-classical monocytes comprising the mature subset [47]. Cord blood PBMC from Tdap-IPV-vaccinated pregnancies contained a higher proportion of classical monocytes and reduced frequency of intermediate monocytes in both resting conditions and following exposure to Tdap-IPV vaccine antigens. The mechanism of this rather striking observation is unclear. Observed differences were not due to *in vitro* adherence of activated monocytes; polypropylene was used throughout for PBMC assays and EDTA used to detach any potential adherent cells. Myeloid cells originate in the fetal circulation as early as three weeks gestation [48] which has been proposed to make them potentially susceptible to reprogramming by the maternal environment [49]. Interestingly, this difference in monocyte distributions between vaccinated and unvaccinated groups was not observed following *B. pertussis* nor LPS exposure, suggesting that any Tdap-IPV-induced differences in the distribution of monocyte populations in infants at birth are likely to be lost upon pathogen encounter. As CD16 is essential for monocyte antibody-dependent cellular cytotoxicity (ADCC) [50] and an increase in the proportion of monocytes lacking CD16 may have implications for the neonatal innate immune response, it would be valuable to monitor these responses across the neonatal period and in infancy to ascertain if indeed this represents a more long-lasting effect on monocyte populations.

Unlike the higher IL-12 whole blood responses in neonates from vaccinated pregnancies, the proportion of IL-12-positive monocytes following *B. pertussis* stimulation were lower, suggesting that IL-12 in the whole blood assay originated from other cellular sources, such as neutrophils. We did not investigate neutrophils by flow cytometry, as they are strongly affected by cryopreservation. The proportion of IFN-γ-positive NK cells was also lower in neonates from vaccinated pregnancies. Innate IL-12 and IFN-γ are important in the immune response to *B. pertussis* infection. Both cytokines help to polarise the T cell response [25] and IFN-γ localises *B. pertussis* to the lungs in animals models [23,24] as well as enhancing the killing of *B. pertussis* by macrophages. Unlike the whole blood assay, the PBMC stimulations lacked plasma (and thus maternal IgG). Several immune cells express receptors for the Fc domain of IgG, and interactions between antibody:antigen complexes with Fc receptors on immune cells modulate the innate and adaptive immune response. For example, during antibody-dependent cellular cytotoxicity (ADCC), cytokine production can be triggered in NK cells through binding of antibody that has opsonised its target to NK cell Fc receptors [51]. Although removal of plasma from the whole blood assay reduced the cytokine response to *B. pertussis*, it was restored by replacement with serum from both Tdap-IPV-vaccinated and unvaccinated pregnancies, suggesting that the differences observed were not solely due to higher levels of maternal anti-pertussis IgG in babies from vaccinated pregnancies.

Beyond exploring the immune response at birth, we studied the response to *B. pertussis* in infants at seven weeks (pre-pertussis immunisation) and five months of age (post-primary immunisation) in whole blood in order to explore the potential for longer-term effects of maternal Tdap-IPV. As was observed at birth, infants from Tdap-IPV-vaccinated pregnancies had increased IL-2 responses at seven weeks of age, compared to infants from unvaccinated pregnancies. As described earlier, IL-2 acts as a T cell growth factor [45,46] and its elevation in infants at seven weeks of age may enhance the expansion and activation of T cells that are primed by subsequent primary pertussis immunisation. However, the clinical consequences in the context of infection or vaccination remains to be established.

During *B. pertussis* infection, T helper cells polarise towards a (Th) 1 cytokine response, [45,46] that enables the killing of bacteria. In contrast, following acellular pertussis vaccination there is the induction of Th2 cytokines, suggesting a skewing of the T cell response [52,53]. In the present study, there were decreases in the cytokine response to *B. pertussis* in infants from Tdap-IPV-vaccinated pregnancies at seven weeks and/or five months of age for IL-4, IL-10 and IL-13, compared to the unvaccinated group. These cytokines are normally associated with Th2 responses, which following acellular pertussis vaccination are not associated with protective immunity against pertussis in animal models [54]. The decrease in IL-10 responses observed in infants at seven weeks and five months of age from Tdap-IPV-vaccinated pregnancies is in direct contrast to the effect seen in the mothers, where Tdap-IPV-vaccination was associated with elevated IL-10 responses to the bacteria. IL-10 primarily acts as an anti-inflammatory cytokine and can inhibit the activity of NK cells, T helper 1 cells and macrophages [55]. IL-10 from innate cells has previously been shown to reduce *B. pertussis*-specific pro-inflammatory responses in infants that received an acellular pertussis vaccine [56]. Innate immune cells have the capacity to secrete IL-10 [25] and given the short incubation period after which supernatants were harvested these are the most likely source. Our data suggests that maternal Tdap-IPV vaccination in pregnancy has a longer lasting effect on the IL-10 response to *B. pertussis* in the offspring, which may play a role in inhibiting pertussis vaccine responses in infants. However, the biological consequences of this small, though significant reduced IL-10 response, remains to be fully determined.

The mechanisms behind our observed effects of maternal pertussis vaccination on infant cell-mediated immunity is currently not known. The developmental origins of health and disease (DoHaD) hypothesis postulates that *in utero* exposure to environmental influences during critical periods of development may impact on short and long-term health of the offspring [57]. Maternal vaccination, which induces a maternal immune response is one such intervention. Maternal cytokines produced in response to vaccination could potentially cross the placenta directly to modify neonatal responses at birth: studies utilising a placental perfusion model to study cytokine transfer have produced conflicting results; one study concluded that maternal IL-6 may cross the placenta [58] whilst another study found that it does not [59]. Both studies only investigated pro-inflammatory cytokines (IL-6, TNF-α, IL-1α/β), and it is not known if other cytokines such as IL-10 can cross into the fetal circulation to modulate the fetal immune system. Furthermore, *in utero* exposure to antigens has been associated with altered immune responses at birth [60] potentially facilitated through passage of antigen-antibody complexes across the placenta [61].

Our study subjects were recruited when maternal vaccination in the UK was recommended between 28–32 weeks gestation [62] which is reflected in our mean of 30.9 weeks gestation (mode 30 weeks) of maternal vaccination across all our subjects. Since April 2016, the vaccine has been recommended to be administered between 16–32 weeks [63] partly due to reports that cord blood antibody levels may be higher when women are vaccinated in the second trimester [64] and to better protect babies that deliver preterm. It would therefore be of interest to determine whether earlier gestation at Tdap-IPV vaccine administration also has a greater effect on offspring cellular immune responses.

Our study has some limitations. The flow cytometry assays were only performed on cord blood due to the sample volume requirement. Therefore, the flow data only reflects the infant immune system at birth, and future studies should investigate whether maternal Tdap-IPV has a longer-lasting effect on infant monocyte populations. Flow cytometry was performed on cryopreserved PBMC, which enabled us to analyse batched vaccinated and unvaccinated samples, reducing technical and reagent variation, but this could have impacted the expression of activation markers and cytokine production. Most of our subjects received Boostrix-IPV® Tdap-IPV vaccination, but some women may have received a different brand (Repevax®). As the vaccine was delivered through primary care at this time, the brand administered was not recorded in hospital records, and so we relied on patient recall. We therefore had some patients (4/52) with unknown vaccine brands, although these were all vaccinated after the switch to Boostrix-IPV®, so it is probable that all of the patients in this study did receive the same vaccine. For these reasons, we were unable to investigate any potential differences in responses to different Tdap-IPV vaccine formulations. As infants also receive their primary immunisations through primary care, we were not able to access information on the brand of vaccine they had received, and hence could not compare responses between infants that had received a primary course of either Pediacel® or Infanrix-IPV-Hib®. Although as of 2017, all infants in the UK are now offered the 6-in-1 vaccine Infanrix hexa® as their primary pertussis vaccine. Further, we did not assess cytokine responses to *B. pertussis* in non-pregnant females, to help elucidate any potential effects of pregnancy on the innate immune response to *B. pertussis*. As pertussis vaccination is recommended during pregnancy by health authorities in the UK, a randomized control trial with a no-vaccine arm was not possible due to ethical reasons. Therefore, unvaccinated pregnant women in this study did not receive Tdap-IPV for a variety of reasons, including through their own choice or because they were unaware of the recommendation, as we have previously described in this population [65].

In conclusion, maternal Tdap-IPV vaccination may be associated with altered monocyte subsets at birth and reduced monocytic IL-12 and NK cell IFN-**γ** responses to *B. pertussis* (in the absence of IgG), pointing towards the intriguing possibility that maternal Tdap-IPV may lead to additional *in utero* modification of the fetal immune system, beyond effects mediated by IgG transfer alone. In the whole blood assay, we conversely saw elevated IL-2 and IL-12 at birth. Importantly, levels of IL-2 remained elevated at seven weeks of age and could therefore play a role in modulation of T cell function in these infants. Other than elevated IL-2, maternal Tdap-IPV otherwise resulted in a reduction in Th2 cytokine responses (IL4, IL-10, IL-13) in blood from older infants, which could play a role in the reduced humoral response that has been observed in infants from Tdap/Tdap-IPV-vaccinated pregnancies. [17], [18], [19], [20] Any clinical relevance of these observations in terms of infant responses to infection or vaccination remains to be established. Given that certain vaccines may also have non-specific effects through epigenetic reprogramming of innate cells [66,67] - a concept termed trained immunity - future studies should investigate whether maternal Tdap-IPV vaccination during pregnancy might alter gene expression in fetal immune cells. Although it is important to note that to date, vaccines shown to demonstrate trained immune effects on the innate immune system have all been live vaccines. In the meantime, vaccination of pregnant women against pertussis has already prevented many cases of pertussis in young infants [10], [11], [12]. These interesting immunological findings need to be interpreted in the context of proven vaccine benefit to mothers and babies.

## 5 Contributors

TR, BH, DD and BK contributed to the conception and design of the work. All authors contributed to the acquisition, analysis, or interpretation of data for the work. TR, BH, DD and BK drafted the manuscript. All authors approved the final version of the article to be published. TR and BH have verified the underlying data. TR, BH and BK are accountable for all aspects of the work in ensuring that questions related to the accuracy or integrity of any part of the work are appropriately investigated and resolved.

## 6 Data sharing statement

Data from this study will be made available to qualified investigators upon reasonable request to the corresponding author.

## Declaration of Competing Interest

No conflicts of interest exist for any of the authors.

## Appendix Supplementary materials

Image, application 1
