# Supplementary material for: Modification of innate immune responses to Bordetella pertussis in babies from pertussis vaccinated pregnancies
Source: eBioMedicine. 2021 Oct 11;72:103612. doi: 10.1016/j.ebiom.2021.103612 (PMC8517834; doi:10.1016/j.ebiom.2021.103612)
Supplement: Supplementary file 1 [file mmc1.docx]

**Caption for supplementary material**

Supplementary Table 1. Antibodies used for identification of innate cell subsets and cytokines.

| **Antibody** | **Clone** | **Volume (μl)** | **RRID** |
| --- | --- | --- | --- |
| Alexa Fluor® 700 anti-human CD3 | SK7 | 2.5 | AB_2563419 |
| PerCP/Cy5.5 anti-human CD19 | HIB19 | 5 | AB_2073119 |
| Brilliant Violet 711™ anti-human CD56 | HCD56 | 2.5 | AB_2562417 |
| Brilliant Violet 421™ anti-human CD107a | H4A3 | 5 | AB_10899581 |
| PE/Cy7 anti-human CD16 | B73.1 | 2.5 | AB_2562950 |
| Brilliant Violet 605™ anti-human CD14 | M5E2 | 1.25 | AB_2563798 |
| APC/Fire™ 750 anti-human HLA-DR | L243 | 5 | AB_2572101 |
| Brilliant Violet 785™ anti-human CD123 | 6H6 | 2.5 | AB_2566448 |
| PE/Dazzle™ 594 anti-human CD11c | 3.9 | 2.5 | AB_2564082 |
| PE anti-human IFN-γ | B27 | 2.5 | AB_315439 |
| APC anti-human IL-10 | JES3-9D7 | 5 | AB_315175 |
| PE anti-human IL-6 | MQ2-13A5 | 2.5 | AB_315154 |
| APC anti-human IL-1β | 8516 | 2.5 | AB_2233622 |
| PE anti-human TNF-α | MAb11 | 2.5 | AB_315260 |
| APC anti-human IL-12 | C11.5 | 5 | AB_315191 |

Supplementary Table 2. Patient information for maternal whole blood samples.

|  | | Unvaccinated, n=10 | Vaccinated, n=11 | p value |
| --- | --- | --- | --- | --- |
| Maternal age, years | | 34 (±5.13) | 36 (±5.47) | p > 0.05 |
| Maternal BMI, kg/m^2^ | | 22.67 (±3.1) | 21.41 (±2.35) | p > 0.05 |
| Ethnicity | **White - British** | 40% (4/10) | 18.2 (2/11) | p > 0.05 |
|  | **Any Other White background** | 30% (3/10) | 54.5% (6/11) |  |
|  | **Mixed - White & Black Caribbean** | - | 9.1% (1/11) |  |
|  | **Chinese** | - | 9.1% (1/11) |  |
|  | **Black/Black British African** | 10% (1/10) | - |  |
|  | **Other** | 20% (2/10) | 9.1% (1/11) |  |
| Pertussis vaccine in childhood | | 90% (9/10) | 90.91% (10/11) | p > 0.05 |
| Tdap-IPV in pregnancy | | - | 100% (11/11) | p **< 0.001** |
| Brand Tdap-IPV vaccine | | - | 100% Boostrix (11/11) |  |
| Weeks gestation at Tdap-IPV (unknown for 3) | | - | 31 (±1.89) | - |
| Influenza vaccine in pregnancy | | 20% (2/10) | 45.5% (5/11) | p > 0.05 |
| Month/year of baby’s birth | | 04/2015 - 12/2016 | 08/2015 – 02/2016 | - |
| Gestation at delivery, weeks | | 40 (±0.99) | 40 (±1.14) | p > 0.05 |
| Parity | | 1.3 ((±1.16) | 0.6 (±0.67) | p > 0.05 |
| Gravidity | | 2.8 (±1.48) | 1.6 (±0.67) | p > 0.05 |
| Female fetal sex | | 70% (7/10) | 27.3% (3/11) | p > 0.05 |
| Birth weight, kg | | 3.5 (±0.44) | 3.6 (±0.42) | p > 0.05 |

Supplementary Table 3. Patient information for cord whole blood samples.

|  | | Unvaccinated, n=10 | Vaccinated, n=11 | p value |
| --- | --- | --- | --- | --- |
| Maternal age, years | | 34 (±4.38) | 36 (±5.6) | p > 0.05 |
| Maternal BMI, kg/m^2^ | | 24.14 (±3.91) | 21.84 (±2.12) | p > 0.05 |
| Ethnicity | **White - British** | 30% (3/10) | 18.2% (2/11) | p > 0.05 |
|  | **Any Other White background** | 40% (4/10) | 54.2% (6/11) |  |
|  | **Chinese** | - | 18.2% (2/11) |  |
|  | **Black/Black British African** | 10% (1/10) | - |  |
|  | **Other** | 20% (2/10) | 9.1% (1/11)_ |  |
| Pertussis vaccination in childhood | | 70% (7/10) | 81.8% (9/11) | p > 0.05 |
| Tdap-IPV in pregnancy | | - | 100% (11/11) | **p < 0.0001** |
| Brand Tdap-IPV vaccine | | - | 100% Boostrix (11/11) | - |
| Weeks gestation at Tdap-IPV (unknown for 2) | | - | 30 (±1.56) | - |
| Influenza vaccine in pregnancy | | 0% (0/10) | 36.4% (4/11) | p > 0.05 |
| Month/year of baby’s birth | | 04/2015 - 02/2017 | 08/2015 - 08/2016 | - |
| Gestation at delivery, weeks | | 40 (±1.06) | 39 (±0.93) | p > 0.05 |
| Parity | | 1.4 (±1.17) | 0.5 (±0.69) | p > 0.05 |
| Gravidity | | 2.9 (±1.45) | 1.5 (±0.69) | p > 0.05 |
| Female fetal sex | | 80% (8/10) | 36.4% (4/11) | p > 0.05 |
| Birth weight, kg | | 3.5 (±0.47) | 3.5 (±0.31) | p > 0.05 |

Supplementary Table 4. Patient information for seven week infant whole blood samples.

|  | | Unvaccinated, n=10 | Vaccinated, n=10 | p value |
| --- | --- | --- | --- | --- |
| Maternal age, years | | 32 (±4.38) | 33 (±2.82) | p > 0.05 |
| Maternal BMI, kg/m^2^ | | 26.47 (±4.82) | 23.49 (±3.94) | p > 0.05 |
| Ethnicity | **White - British** | 40% (4/10) | 30% (3/10) | p > 0.05 |
|  | **White- Irish** | - | - |  |
|  | **Any Other White background** | 20% (1/10) | 30% (3/10) |  |
|  | **Mixed - White & Black Caribbean** | - | 20% (1/10) |  |
|  | **Mixed - White & Asian** | - | 10% (1/10) |  |
|  | **Asian/ Asian British - Indian** | 20% (1/10) | - |  |
|  | **Black/Black British African** | - | 10% (1/10) |  |
|  | **Other** | 20% (1/10) | - |  |
| Pertussis vaccination in childhood | | 70% (7/10) | 80% (8/10) | p > 0.05 |
| Tdap-IPV in pregnancy | | - | 100% (10/10) | **p < 0.0001** |
| Brand Tdap-IPV vaccine | | - | 100% Boostrix (11/11) |  |
| Weeks gestation at Tdap-IPV | | - | 32 (±2.67) | - |
| Influenza vaccine in pregnancy | | 0% (0/10) | 100% (10/10) | **p = 0.024** |
| Month/year of baby’s birth | | 03/2015 - 07.2016 | 07/2015 - 01.2016 | - |
| Gestation at delivery, weeks | | 40 (±1.33) | 41 (±0.82) | p > 0.05 |
| Parity | | 1.0 (±1.15) | 0.3 (±0.48) | p > 0.05 |
| Gravidity | | 2.4 (±1.43) | 1.4 (±0.70) | p > 0.05 |
| Female fetal sex | | 70% (7/10) | 40% (4/10) | p > 0.05 |
| Birth weight, kg | | 3.4 (±0.47) | 3.6 (±0.45) | p > 0.05 |

Supplementary Table 5. Patient information for five month infant whole blood samples.

|  | | Unvaccinated, n=7 | Vaccinated, n=10 | p value |
| --- | --- | --- | --- | --- |
| Maternal age, years | | 31 (±5.36) | 34 (±4.36) | p > 0.05 |
| Maternal BMI, kg/m^2^ | | 25.43 (±5.19) | 23.46 (±2.54) | p > 0.05 |
| Ethnicity | **White - British** | 42.9% (3/7) | 30% (3/10) | p > 0.05 |
|  | **White- Irish** | - | 10% (1/10) |  |
|  | **Any Other White background** | 42.9% (3/7) | 20% (2/10) |  |
|  | **Mixed - White & Black Caribbean** | - | 10% (1/10) |  |
|  | **Mixed - White & Asian** | - | 10% (1/10) |  |
|  | **Other** | 14.3% (1/7) | 10% (1/10) |  |
| Pertussis vaccination in childhood | | 85.7% (6/7) | 90% (9/10) | p > 0.05 |
| Tdap-IPV in pregnancy | | - | 100% (10/10) | **p < 0.0001** |
| Brand Tdap-IPV vaccine | | - | 70% Boostrix (7/10), 3 unknown |  |
| Weeks gestation at Tdap-IPV | | - | 32 (±3.60) | - |
| Influenza vaccine in pregnancy | | 14.3% (1/7) | 62.5% (5/8) | p > 0.05 |
| Month/year of baby’s birth | | 02/2015 - 06/2016 | 04/2015 - 09/2015 | - |
| Gestation at delivery, weeks | | 40 (±1.21) | 41 (±0.92) | p > 0.05 |
| Parity | | 0.6 (±0.79) | 0.1 (±0.32) | p > 0.05 |
| Gravidity | | 2.0 (±1.53) | 1.1 (±0.32) | p > 0.05 |
| Female fetal sex | | 42.9% (3/7) | 50% (5/10) | p > 0.05 |
| Birth weight, kg | | 3.6 (±0.50) | 3.6 (±0.40) | p > 0.05 |

Supplementary Table 6. Patient information for cord PBMC samples.

|  | | Unvaccinated, n=10 | Vaccinated, n=10 | p value |
| --- | --- | --- | --- | --- |
| Maternal age, years | | 35 (±4.83) | 36 (±2.83) | p > 0.05 |
| Maternal BMI, kg/m^2^ | | 24.46 (±3.75) | 26.11 (±3.84) | p > 0.05 |
| Ethnicity | **White - British** | - | 20% (2/10) | p > 0.05 |
|  | **White- Irish** | - | 10% (1/10) |  |
|  | **Any Other White background** | 40% (4/10) | - |  |
|  | **Mixed - White & Black Caribbean** | - | 10% (1/10) |  |
|  | **Black / Black British - Caribbean** | 10% (1/10) | - |  |
|  | **Mixed - White & Asian** | - | 10% (1/10) |  |
|  | **Any other Asian background** | 20% (2/10) | - |  |
|  | **Black/Black British African** | 10% (1/10) | - |  |
|  | **Other** | 20% (2/10) | 50% (5/10) |  |
| Pertussis vaccination in childhood | | 30% (3/10) | 20% (2/10) | p > 0.05 |
| Tdap-IPV in pregnancy | | - | 100% (10/10) | **p < 0.0001** |
| Brand Tdap-IPV vaccine | | - | 90% Boostrix (9/10), 1 unknown | - |
| Weeks gestation at Tdap-IPV (unknown for 1) | | - | 31 (±4.70) | - |
| Influenza vaccine in pregnancy | | 20% (2/10) | 50% (5/10) | p > 0.05 |
| Month/year of baby’s birth | | 10/2014 - 01/2019 | 12/2014 - 09/2018 | - |
| Gestation at delivery, weeks | | 39 (±0.63) | 39 (±0.82) | p > 0.05 |
| Parity | | 1.2 (±0.79) | 0.8 (±0.63) | p > 0.05 |
| Gravidity | | 3.0 (±1.89) | 2.2 (±0.79) | p > 0.05 |
| Female fetal sex | | 30% (3/10) | 40% (4/10) | p > 0.05 |
| Birth weight, kg | | 3.5 (±0.52) | 3.3 (±0.24) | p > 0.05 |


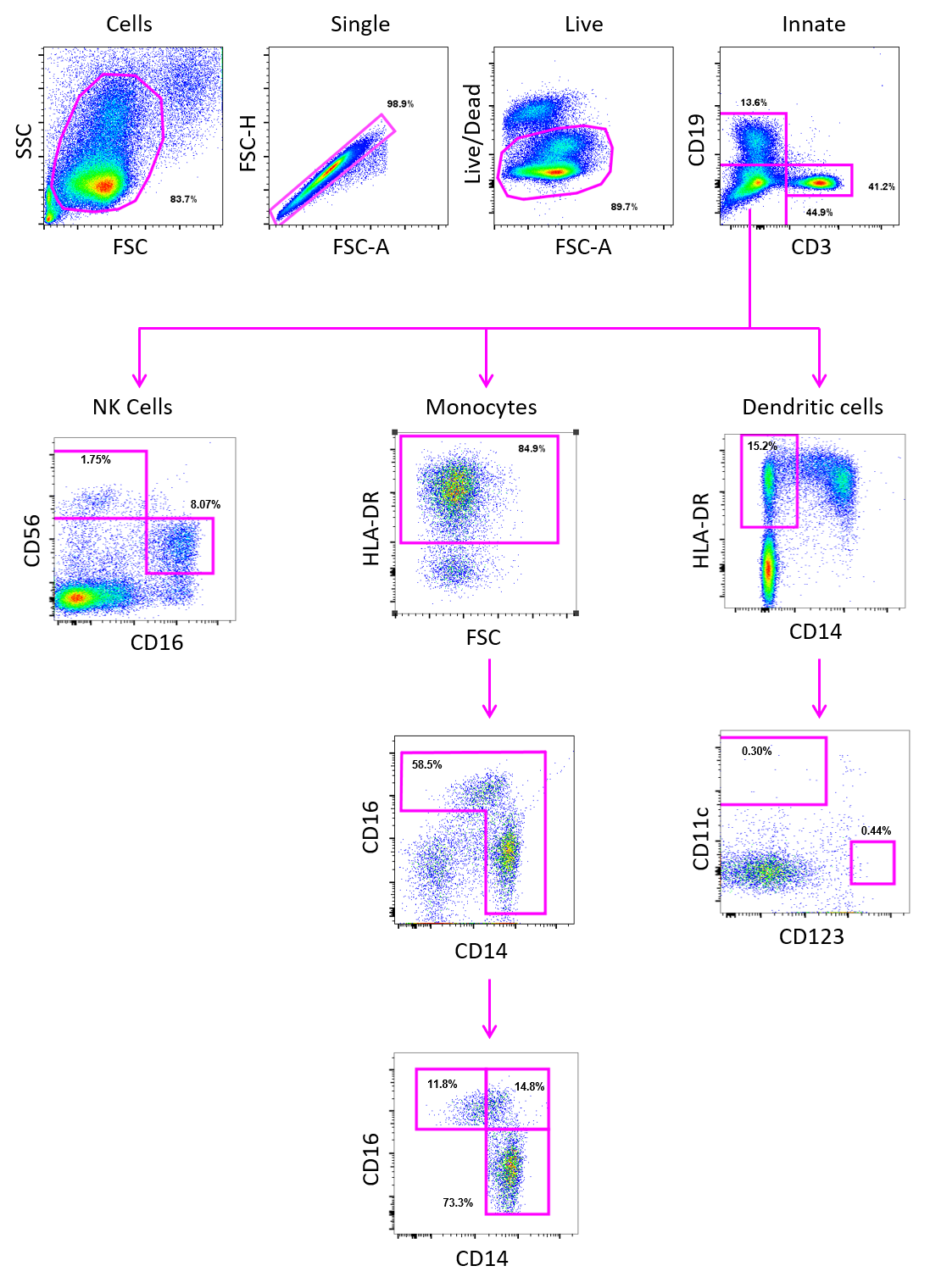


Supplementary Figure 1. Gating strategy to identify innate cell populations.


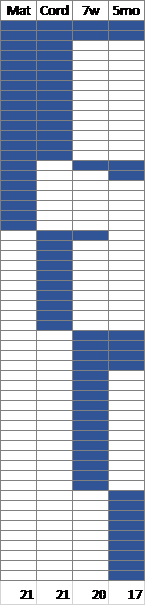


Supplementary Figure 2. Study subjects included at each time point of the whole blood assay.


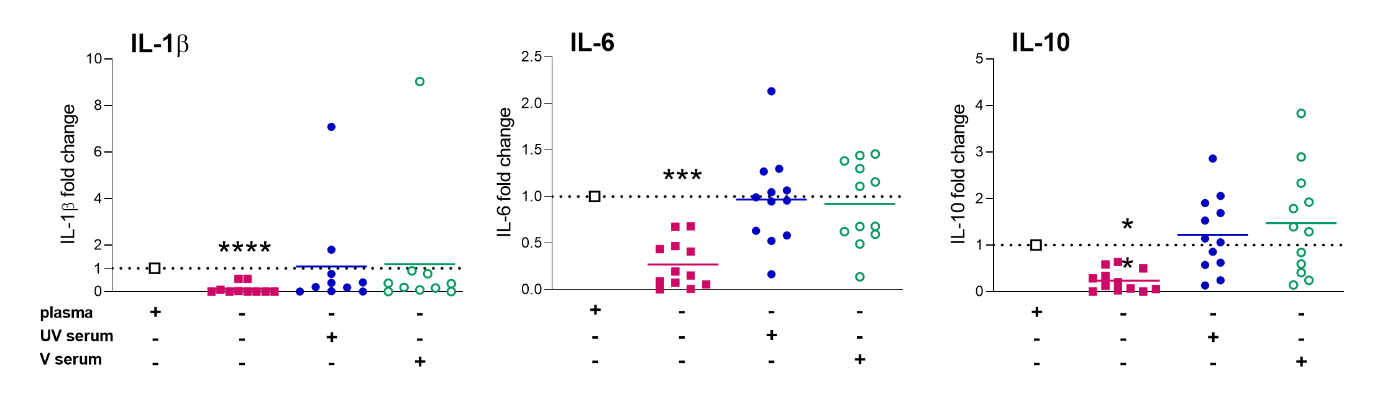


Supplementary Figure 3. Contribution of plasma factors to cord blood cytokine response.


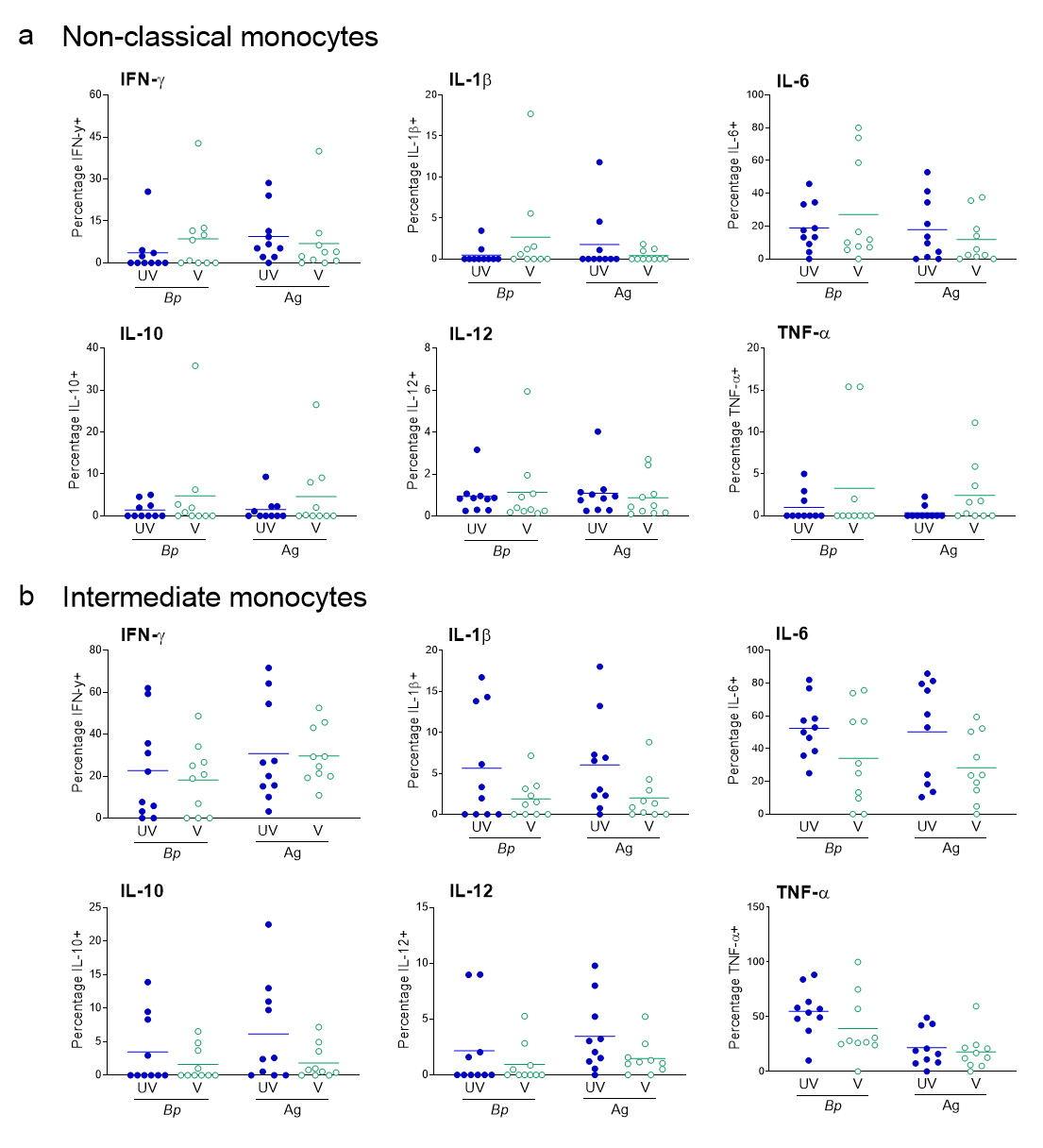


Supplementary Figure 4. Cytokine levels in cord intermediate and non-classical monocytes.


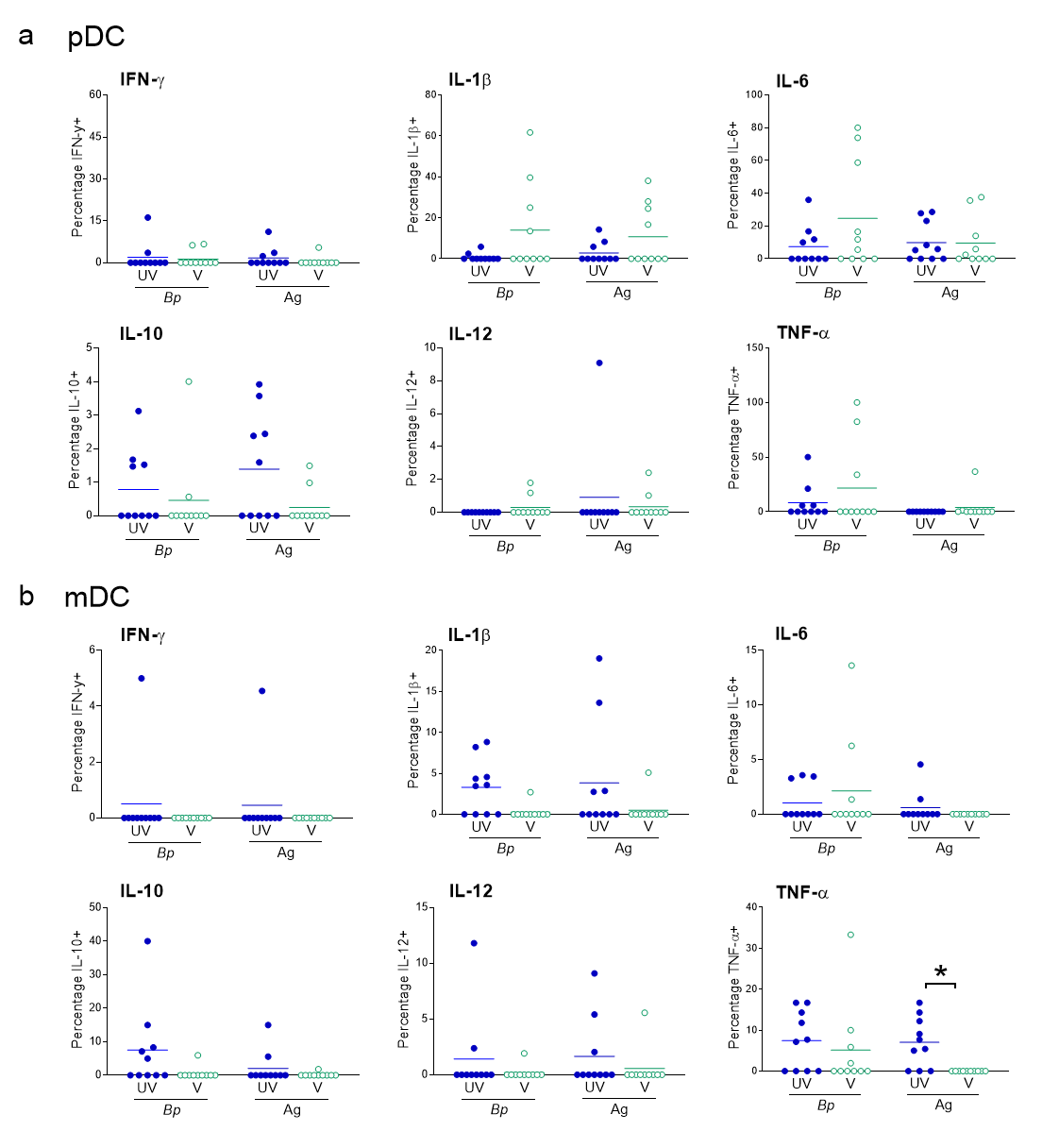


Supplementary Figure 5. Cytokine levels in cord myeloid and plasmacytoid dendritic cells.


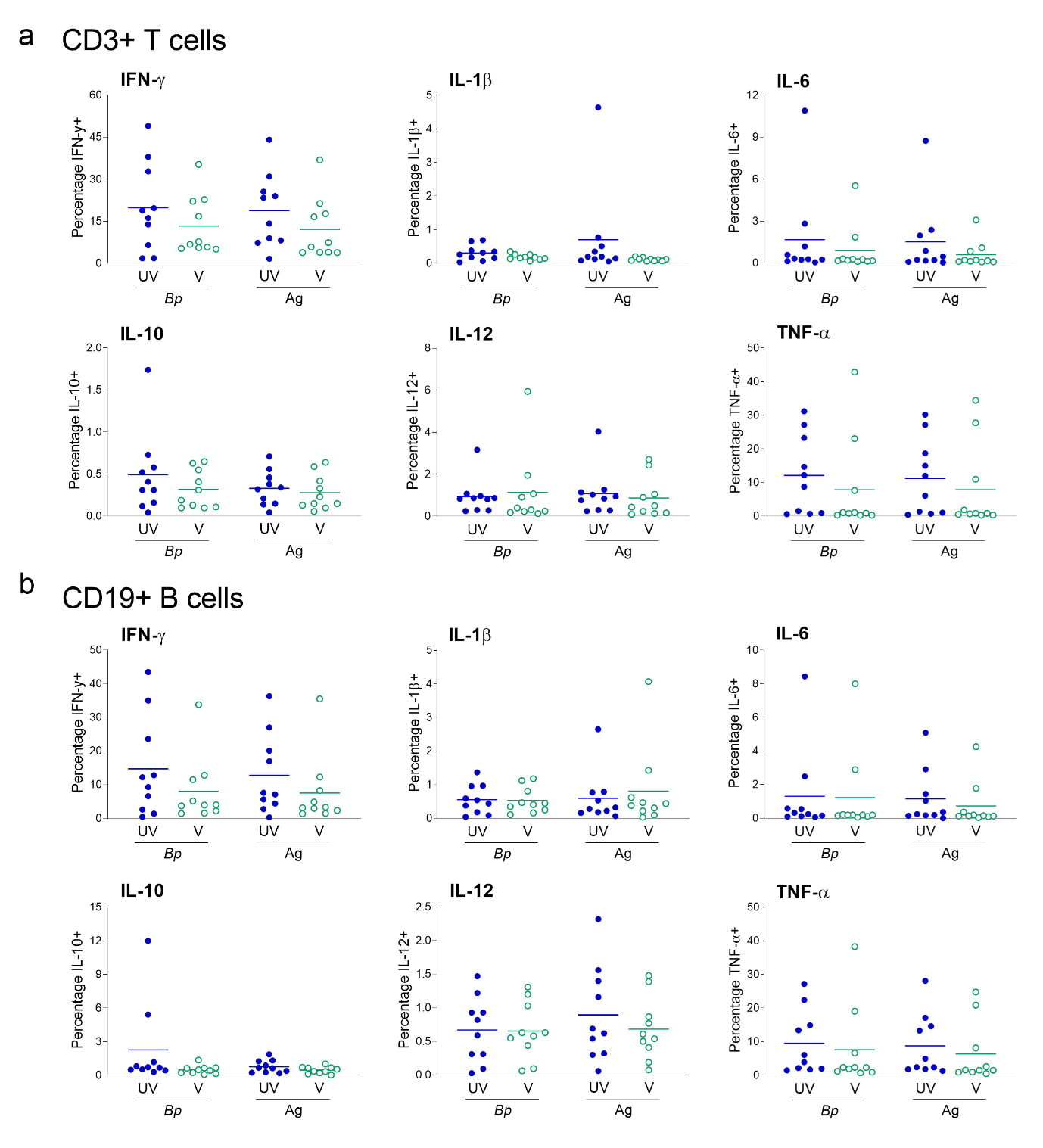


Supplementary Figure 6. Cytokine levels in cord T and B cells.
